# A Facile Method to Synthesize b-Oriented Silicalite-1 Thin Film

**DOI:** 10.3390/membranes12050520

**Published:** 2022-05-13

**Authors:** Montree Thongkam, Somsak Woramongkolchai, Sairoong Saowsupa, Pesak Rungrojchaipon

**Affiliations:** 1 Department of Chemistry, Faculty of Science, King Mongkut’s Institute of Technology Ladkrabang, Ladkrabang, Bangkok 10520, Thailand; montree.th@kmitl.ac.th (M.T.); somsak.wo@kmitl.ac.th (S.W.); 2 Department of Curriculum and Instruction, Faculty of Education, Chulalongkorn University, Bangkok 10330, Thailand; sairoong.s@chula.ac.th

**Keywords:** silicalite-1, membrane, SDA, hydrothermal, dip-coating method

## Abstract

Silicalite-1 thin film was prepared with the following batch composition—3TPAOH:25TEOS:1450H_2_O:100EtOH—and synthesized using the hydrothermal technique. Silicalite-1 colloidal crystals were successfully coated on the surface of the silica substrate by the dip-coating method. The investigation of silicalite-1 thin film with organic structure-directing agents (SDA), using a seeding technique with various colloidal seed concentrations, number of dip-coating steps, and crystallization time, were systematically discussed and obtained interesting results. Silicalite-1 powder and Silicalite-1 membrane, the patterns of which showed a unique characteristic crystallography of MFI topology, were characterized by XRD, which indicated the preferred orientation along the *b*-axis perpendicular to the substrate surface. The morphology and crystal size aspect of Silicalite-1 were also examined by a scanning electron microscope (SEM).

## 1. Introduction

The zeolite thin film/membranes are among inorganic membranes which consist of hydrated crystalline aluminosilicates with open three-dimensional framework structures, made up of SiO_4_ and AlO_4_ tetrahedrons linked by sharing their oxygen atom to form regular intracrystalline cavities and channels of atomic dimensions. The research of zeolite thin film/membrane is an attractive topic of inorganic membrane technology due to their various applications, such as gas or liquid separation [1,2,3,4,5], chemical sensors [6,7,8], catalytic membrane [9,10], dielectric thin films [11,12], corrosion-resistant coatings [12,13] and biological application [14].

Typically, zeolite thin film/membranes are prepared by hydrothermal methods of gel containing silica, alumina, cation, organic template and water, which normally are described as a silicate and aluminate solution. The membrane is usually grown on porous supports, with or without a previous seeding step, and the seeding process of the supports leads to a higher reproducibility in the synthesis of the zeolite membranes. There are several techniques to synthesize zeolite thin film/membrane and different methods of zeolite membrane fabrication have been reported, such as one-step in situ hydrothermal synthesis [15,16,17], dry gel conversion [18,19] and seeded thin film/membrane synthesis [20,21]. The seeding technique has been established as a robust and reliable means of synthesis of thin molecular sieve layers. The nucleation and crystal growth mechanism are individually allowed and separately performed. Consequently, the support for seeding has less influence on the surface chemistry, resulting in improved reproducibility, and the detrimental sedimentation of bulk phase crystals can easily be avoided. The ex-situ of crystallized seeds are mostly used for the synthesis of the zeolite thin film/membrane. The seeding particles are attached to the support surface; it can also be directly crystallized onto the support, e.g., using slip casting [22], electrostatic attraction [23], dip coating [24,25] and pulsed laser ablation [26].

Silicalite-1 has a high temperature resistance, as well as strong hydrophobic and oil-wet properties, and organic molecules such as arenes, short-chain alkanes and polyhydric alcohols can be absorbed by silicalite-1 [27,28]. Therefore, this work mostly focuses on the MFI-type zeolite ZSM-5, particularly its aluminum-free analogue, silicalite-1—because of its pore structure, like many industrially important molecules—thus, contributing its potential as several different types of devices, as mentioned above. In addition, we have attempted to investigate the synthesis of silicalite-1 thin film with organic structure-directing agents (SDA); in addition, the use of a seeding technique with various colloidal seed concentrations, dip-coating times repetition, and crystallization time were systematically investigated. The synthesis of powder silicalite-1 as colloidal seed and silicalite-1 thin film was characterized by using Fourier-transform infrared spectroscopy (FTIR), X-ray diffraction (XRD), and scanning electron microscopy (SEM).

## 2. Experimental Methods

### 2.1. Chemicals

The silicon source used was tetraethyl orthosilicate (TEOS, <98%, GC, Merck); tetrapropylammonium hydroxide (TPAOH or TPA, 1.0 M in water, Sigma) was used as the structure-directing agent and ethanol (EtOH, ≥99.5%, Merck). Double-distilled water was used in all cases. All chemicals were used as received without further purification.

### 2.2. Synthesis of Seed MFI (Silicalite-1)

The seed silicalite-1 was prepared from a hydrothermal synthesis of the molar composition of 9TPAOH:25TEOS:480H_2_O:100EtOH. First, the structure-directing solution of 27 mL of 1 M TPAOH was mixed with 27 g of H_2_O, and 15 g of ethanol (EtOH), then stirred for 5 min. The TEOS solution of 15 g was added dropwise into an aqueous TPAOH solution and vigorously stirred. Then, the solution was transferred into the Teflon-lined autoclave and heated to 100 °C for 1 d.

### 2.3. Preparation of Silicon as a Substrate

A silicon substrate in the form of a silicon wafer was cleaned with acetone in an ultrasonic bath for 5 min. Then, the silicon substrate was boiled in the solution with the composition by a volume of 5H_2_O:1H_2_O_2_ (30 wt%): 1NH_3_ (25 wt%) for 5 min, followed by the composition by a volume of 6H_2_O:1H_2_O_2_ (30 wt%):1HCl (37 wt%) for 5 min; at each cleaning step, the substrate was rinsed with distilled water.

### 2.4. Dip Coating of Clean Silicon Substrate into Colloidal Seed Silicalite-1

**Figure 1:**
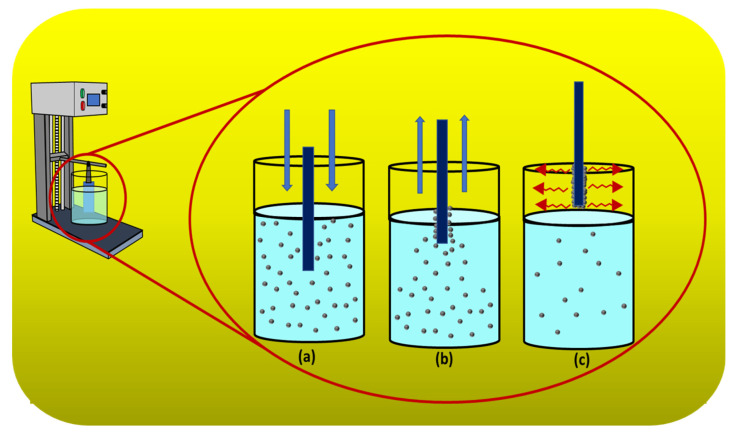
Schematic diagram of dip-coating process. (**a**) Dipping. (**b**) Withdrawing and deposition. (**c**) Solvent evaporation and coating.

A clean silicon substrate was dipped into a colloidal of 0.2, 0.4, and 0.8 g of silicalite-1 in 20 mL ethanol and 0.1 g of cetyltrimethylammonium bromide (CTAB) or without CTAB. The number of dip coat was applied from 1, 3, 5, 7 and 9 for each colloidal concentration. Between each number of coating step, the substrate was dried with nitrogen gas. The substrate with adsorbed seed silicalite-1 monolayer or multilayer was heated to 500 °C in atmosphere for 3 h. The dip-coating process on the substrate is shown in Figure 1. The process was applied by using a dipping machine with a slow dipping rate. The substrate was dipped into the sol precursor. Then, the substrate was withdrawn, and the sol precursor was deposited. Finally, the solvent on the substrate was evaporated; therefore, the coating process was initially recognized, as shown in Figure 1a–c, respectively.

### 2.5. Synthesis of Silicalite-1 Thin Film on Silicon Substrate

The silicalite-1 thin film on silicon substrate was prepared from a hydrothermal synthesis of the molar composition of 3TPAOH:25TEOS:1450H_2_O:100EtOH. First, the structure-directing solution of 3 mL of 1 M TPAOH was mixed with 80 g of H_2_O and 15 g of ethanol, then stirred for 5 min. The 15 g of TEOS was drop wised into an aqueous TPAOH solution and vigorously stirred. Then, the solution was transferred into the Teflon-lined autoclave that contained seeding silicon substrate, and heated to 100 °C for 1 d. The product was washed several times with deionized (DI) water and dried in air. The thin film was calcined at 500 °C with a heating rate of 0.2 °C/min in order to remove any organic SDA in the porous channel.

### 2.6. Characterization

Powder X-ray diffraction data were collected on Scintag XDS 2000 automated powder diffractometers equipped with Cu K_α_ radiation sources (λ = 1.5406 Å). The diffractometers were also equipped with Peltier- or liquid-nitrogen-cooled germanium detectors. A randomly oriented powder sample was analyzed by placing it on top of a stainless-steel or aluminum sample holder. The infrared spectrum was obtained by plotting the transmittance versus the wavenumber. The instrument used in this research was a Galaxy FTIR 5000 series spectrometer. The data were collected by transmittance in a range of 4000–400 cm^−1^ (wavenumbers) at an ambient temperature. The sample was prepared by grinding a small amount of each compound with KBr and pressed into a transparent pellet. The thin film sample was measured directly by using a typical sample holder. A scanning electron microscope (SEM) JEOL JSM-6330 F equipped with EDS was used to determine the composition of samples and particle analysis. The samples were made of a non-metal material; therefore, they had to be coated with a conductive material. This was carried out by using a gold-sputtering coater which made them compatible with SEM.

## 3. Result and Discussion

**Figure 2:**
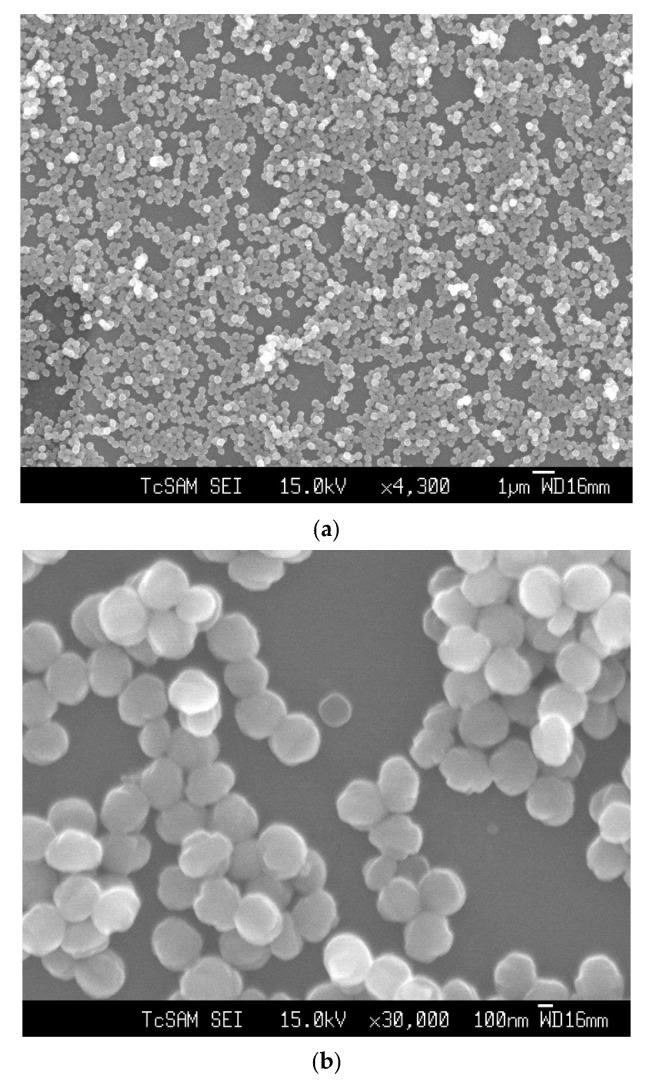
SEM images of seed silicalite-1 at (**a**) low magnification and (**b**) high magnification.

**Figure 3:**
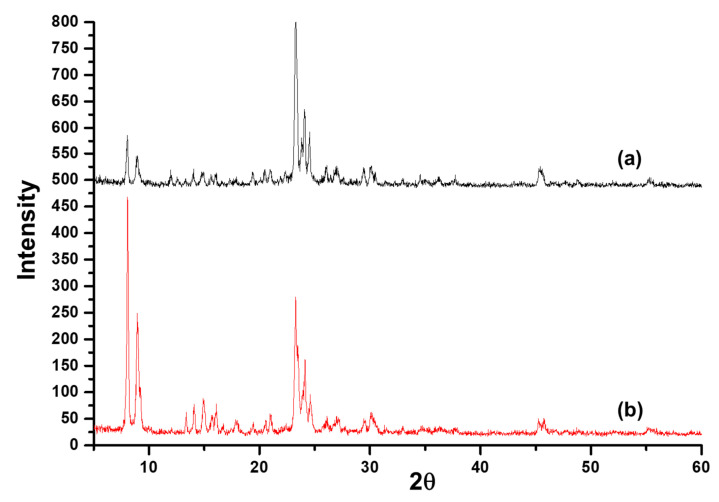
XRD of (**a**) as-synthesized silicalite-1 powder and (**b**) calcined silicalite-1 powder.

Figure 2 shows the SEM image of an as-synthesized nanoparticle of silicalite-1 with (a) low and (b) high magnification, respectively, and a particle size between 100 and 200 nm. These seed silicalite-1 were used as colloidal seed silicalite-1 for the synthesis of silicalite-1 thin film. Typically, a method to obtain a smaller seed silicalite-1, the synthesis of seed silicalite-1, could be synthesized by using a lower temperature and collecting the product by using ultrahigh speed centrifuge. However, temperature effects not only the size of the silicalite crystal, but also the morphology of crystals, and the aspect ratio of silicalite crystals increases with temperature [29,30]. The growth rate of each crystal’s face has been studied, especially for the case of silicalites which corresponds to different activation energies, as mentioned in the literature [30,31]. Figure 3 shows an XRD of as-synthesized (as-syn) powder and calcined powder of silicalite-1. XRD is typical for randomly oriented silicalite-1 and showed that the product consisted of well-crystallized TPA-silicalite-1 designated for as-synthesized silicalite-1 powder (Figure 3a) and calcined silicalite-1 powder (Figure 3b). The degree of crystallinity was determined by Origin 2020 using a peak analyzer and integration method. The percentage crystallinity of as-synthesized silicalite-1 powder and calcined silicalite-1 powder are 79.82%, and 98.79%, respectively. It is indicated that after the calcination of as-synthesized silicalite-1 powder, the designated as-calcined silicalite-1 and the degree of crystallinity increased.

**Figure 4:**
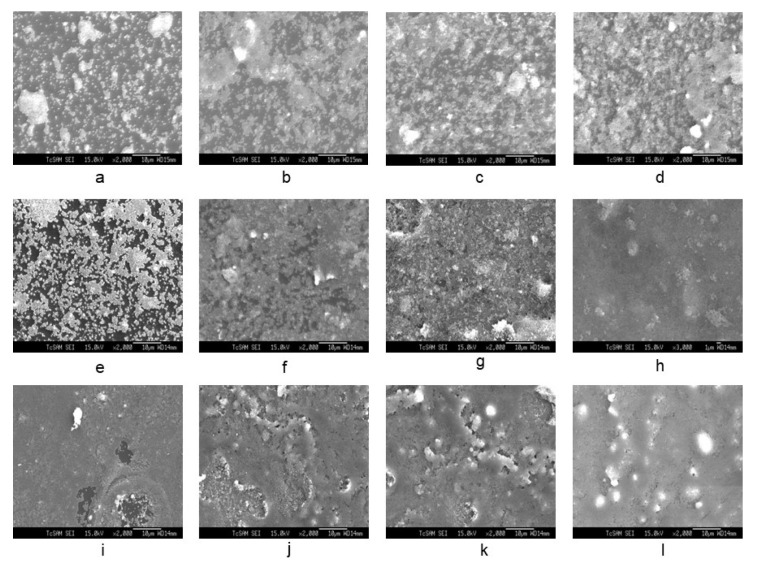
SEM image of seed silicalite-1 on silicon substrate at varying colloidal seed concentrations and number of dip coating (**a**–**d**). The 1 wt% seed silicalite-1, 1 wt% CTAB and 20 mL ETOH with coating for 1, 3, 5 and 7 times (**e**–**h**). The 4 wt% seed silicalite-1, 1 wt% CTAB and 20 mL ETOH with coating for 1, 3, 5 and 7 times (**i**–**l**). The 4 wt% seed silicalite-1 and 20 mL ETOH with coating for 3, 5, 7 and 9 times, respectively.

**Figure 5:**
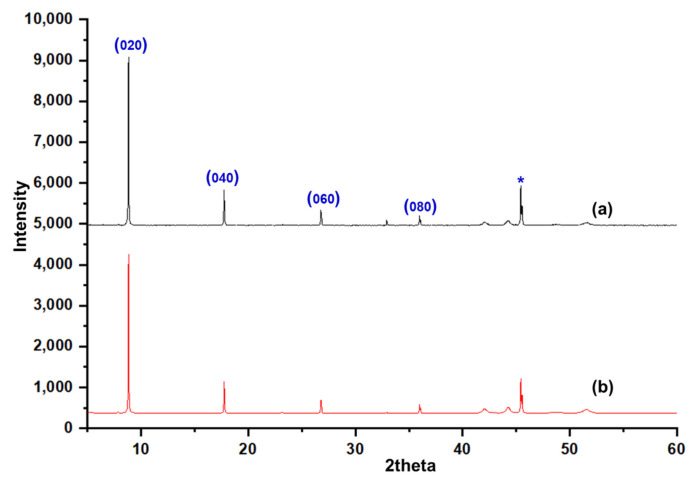
XRD of (**a**) as-syn silicalite-1 thin film, (**b**) calcined silicalite-1 thin film and reflection marked by (*) correspond to the silicon substrate.

**Figure 6:**
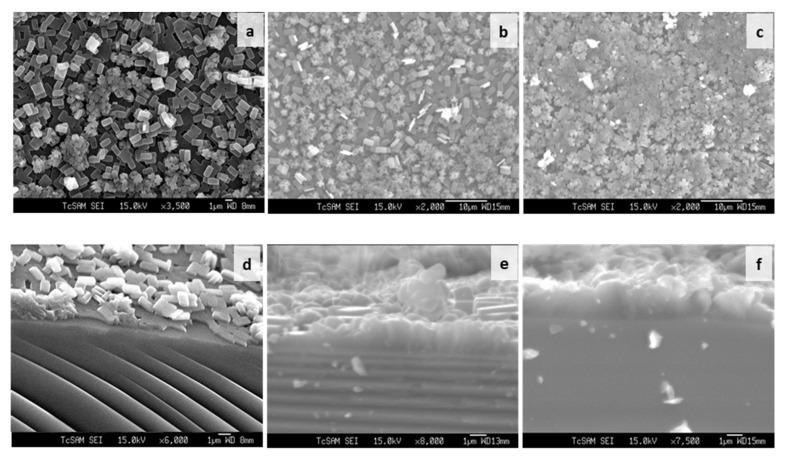
SEM images of silicalite-1 thin-film surface (**a**–**c**) and cross section (**d**–**f**) synthesized at 100 °C after 1 day (**a**,**d**), 3 days (**b**,**e**), and 5 days (**c**,**f**) of crystallization.

The SEM image of Figure 4a–l shows the seeding of colloidal silicalite-1 on a silicon wafer with varying colloidal seed concentrations (1 wt%, 2 wt% and 4 wt% of solution, respectively) and various numbers of dip coating. The cationic surfactant cetyltrimethylammonium ion (CTA^+^) was used to enhance the suspension of uniform silicalite-1 colloidal coated with CTAB. In Figure 4a–h, the dip-coating process of colloidal seed with 1% CTAB has shown that the deposition of a seeding silicalite-1 on silicon substrates appears to be increasingly more aggregation-prone than without CTAB. The aggregation of silicalite-1 colloidal could be affected from the critical micelle concentration (CMC), which is the concentration of surfactants above which micelles are spontaneously formed [32,33,34]. However, the deposition of the seeding of silicalite-1 on silicon substrates without CTAB for Figure 4i–l indicated that the seed coverage fully coated almost 90% of the substrate surface and showed a small aggregation of silicalite-1 colloidal on the silicon surface. Moreover, it is clearly shown that, when dip-coating times increase to three times in Figure 4i, the substrate was fully covered by silicalite-1 seed; however, this was not the case in the situation shown in the Figure 4f. Thus, in this experiment, the preference of dip-coating repetition times and colloidal seed concentration will be used according to the conditions, as shown in Figure 4i. Figure 5 shows the XRD of as-syn silicalite-1 membrane and calcined silicalite-1, showing that they have the same crystallographic preferred orientation. The XRD patterns of Figure 5 show that the silicalite-1 membrane gives relatively intense single (0k0) peaks; this indicates that the silicalite-1 crystals have their *b*-axis perpendicular to the substrate surface [35,36,37]. Weijiong et al. [38] showed that the preferred orientation from SEM images is only possible when the crystals have developed a typical morphology. Moreover, SEM images of the silicalite-1 membrane on the surface of the substrate (Figure 6a) show that the silicalite-1 crystals on the silicon substrate have a typical hexagonal prismatic shape, or more commonly referred to as a coffin shape, with the order of crystal dimensions being L_c_ > L_a_ > L_b_ (where L_i_ indicates crystal size along i-axis); they show good intergrowth behavior.

The seeded substrates were hydrothermally treated in the synthesis seed solution for the desired periods of time. Figure 6d–f show SEM images of the silicalite-1 thin-film cross section synthesized at 100 °C after 1 day, 3 days and 5 days of crystallization. The film thickness was estimated from SEM images. However, the film thickness that was determined from the SEM side view images was only an estimate measurement, as some seed depositions do not form a dense film. As shown in Figure 6a, the SEM side view image shows the first layer and second layer from the nucleation growth and crystal growth mechanism, respectively. The first layer is approximately the same thickness as the colloidal seed size. As the synthesis time increased, the silicalite-1 thin films appeared to increase their thickness. The silicalite-1 thin films of Figure 6d–f are estimated to be approximately 0.5, 1.0 and 2.0 µm-thick, respectively. The surface morphology of the silicalite-1 thin film became rougher at a longer crystallization time, while the surface morphology of the substrates covered by silicalite-1 crystals turned into a more random arrangement. When the silicalite-1 thin film was grown to a thicker layer (Figure 6b,c), the surface of the silicalite-1 thin film became more uneven and rough, which was probably caused by the stacking and tilting of the silicalite-1 crystals.

**Figure 7:**
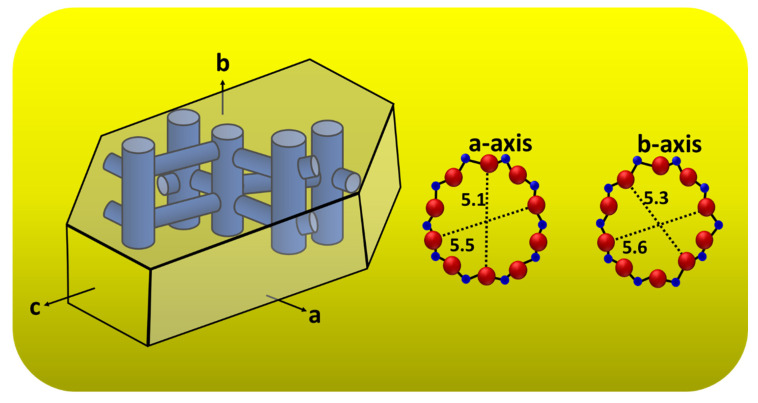
Schematic diagram of an MFI crystal and overview of the channel system of a-axis and b-axis.

The majority of silicalite-1 crystals are oriented with the *b*-axis perpendicular to the substrate surface, which is expected since the (010) face is the largest face of the mother crystal, as shown in Figure 7. These correspond to the XRD results, as shown in Figure 5; the b-oriented film and only (0k0) reflections were observed, confirming that the b-axis of the MFI crystals is perpendicular to the substrate surface. Moreover, Figure 7 shows a schematic diagram of an MFI crystal and an overview of the channel system, which shows that the straight channels are running along the b-axis in the crystal and have a dimension of 5.6 × 5.3 Å. The sinusoidal channels are running along the a-axis within the crystal and have a dimension of 5.5 × 5.1 Å. The calculated data of crystallographic parameter are shown in Table 1. The structural parameters were compared to the literature data of the other reports [39,40], and the present research was studied and discussed. From Table 1, we found that the exposure surface areas of the synthesized silicalite-1 from this research is 65.6%; the result is similar to both samples in the literature—namely, Z-cS^2^ and Z-cM^2^ [40]—which reported 63.0% and 67.2%, respectively. However, the exposure surface areas of another study [39] has shown a slightly different result; in comparison to S1 (this study), it can be indicated that the *b*-axis oriented perpendicular to the crystal surface was preferred for S1. S1 used SDA (TPA), the same as the sample used in Ref. [36] and indicated the case for a better SDA fit in the straight channel, agreeing with the enhancement in growth along the *b*-axis relative to the growth along the *a*-axis [40]. Additionally, it can be observed that as the exposed degree of [010] plane increased resulting in the more of the percentage of straight channels are revealed. Therefore, controlling the membrane morphology of silicalite-1 enabled us to alter the designated function of the MFI membrane for a higher performance, such as the separation ability, adsorption or diffusion properties, and shape-selectivity of catalysis (see Figure S1 for the FTIR of silicalite-1 on silicon substrate).

## 4. Conclusions

The synthesis of colloidal silicalite-1 gel and silicalite-1 thin film were successfully prepared using a simple secondary growth method with the following batch composition: 3TPAOH:25TEOS:1450H_2_O:100EtOH. Moreover, the silicalite-1 colloidal crystals were fully coated on the surface of the silica substrate using the dip-coating method and synthesized using the hydrothermal technique. The investigation of silicalite-1 thin film with organic structure-directing agents (SDA) and the use of the seeding technique with various colloidal seed concentrations, dip-coating times repetition, and crystallization time, were systematically discussed. XRD and SEM have shown interesting results, such as crystallographic parameters, thin-film/membrane thickness and controlled crystal face aspect ratio, which can be applied for the fabrication of zeolite thin-film/membrane and is beneficial at both industrial and academic levels.

## Figures and Tables

**Table 1:** Comparison of morphology parameters of as-synthesized silicalite-1 in this work and the samples reported in the literature.

Samples	L_a_ [nm]	L_b_ [nm]	L_c_ [nm]	[010] Exposure Degree [%] ^1^	L_c_/L_a_	L_c_/L_b_	L_a_/L_b_
S1 (this study)	641	182	1274	65.6	1.98	7.04	3.52
Z-cS ^2^	248	100	548	63.0	2.21	5.48	2.48
Z-cM ^2^	246	97	970	67.2	3.94	10.00	2.53
Z-cL ^2^	249	98	1530	68.9	6.14	15.61	2.54
Seed ^3^	300	200	400	41.4	1.33	2.00	1.50
nonSeed ^3^	10,500	5800	14,100	46.1	1.34	2.43	1.81

^1^ Calculated by the exposure surface areas of various crystal planes. d[010] = S[010]/(S[010] + S[100] + S[101]). ^2^ Ref. [40]. ^3^ Ref. [39].

## Data Availability

The data suporting of this study are available from the corresponding author P.R. on reasonable request.

## Supplementary Materials

The following supporting information can be downloaded at: https://www.mdpi.com/article/10.3390/membranes12050520/s1, Figure S1. FTIR of silicalite-1 on silicon substrate (a). silicalite-1 membrane (c). silicalite-1 powder.
