# Supplementary material for: A Facile Method to Synthesize b-Oriented Silicalite-1 Thin Film
Source: Membranes (Basel). 2022 May 13;12(5):520. doi: 10.3390/membranes12050520 (PMC9147600; doi:10.3390/membranes12050520)
Supplement: Supplementary file 1 [file membranes-12-00520-s001.zip › membranes-1712802-supplementary.pdf]

Supporting Information (SI)

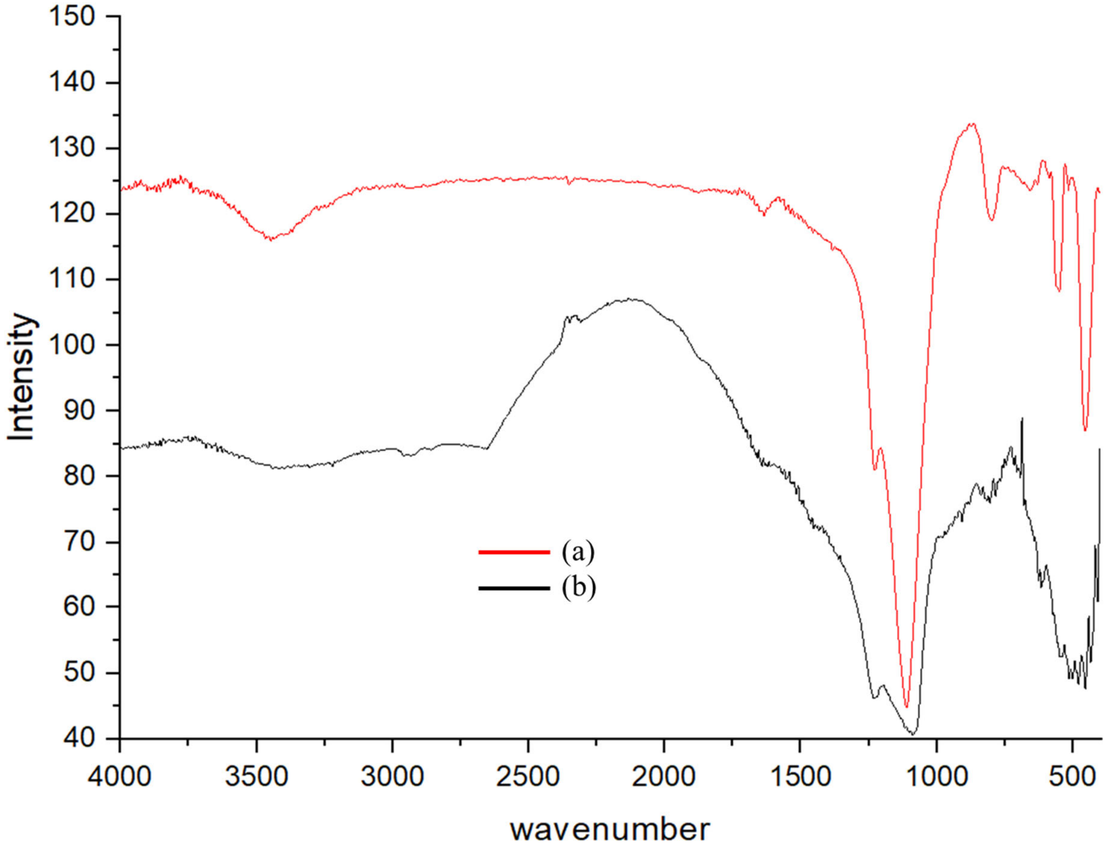

Figure S1. FTIR of silicalite-1 on silicon substrate (a). silicalite-1 membrane (c). silicalite-1 powder.
